# Transgenic Expression of P1A Induced Thymic Tumor: A Role for Onco-Fetal Antigens in Tumorigenesis

**DOI:** 10.1371/journal.pone.0013439

**Published:** 2010-10-15

**Authors:** Chi-Shan Li, Chong Chen, Pan Zheng, Yang Liu

**Affiliations:** 1 Division of Immunotherapy, Section of General Surgery, Department of Surgery, School of Medicine, University of Michigan, Ann Arbor, Michigan, United States of America; 2 Division of Molecular Medicine and Genetics, Department of Internal Medicine, School of Medicine, University of Michigan, Ann Arbor, Michigan, United States of America; 3 Program of Molecular Mechanism of Diseases and Comprehensive Cancer Center, School of Medicine, University of Michigan, Ann Arbor, Michigan, United States of America; 4 Department of Pathology, School of Medicine, University of Michigan, Ann Arbor, Michigan, United States of America; 5 Toxicology Program, Department of Environmental Health Sciences, School of Public Health, University of Michigan, Ann Arbor, Michigan, United States of America; Ohio State University, United States of America

## Abstract

P1A is the first known tumor rejection antigen. It is expressed in embryonic stem cells and multiple tumors but is silent in adult tissues except for the testis and placenta. Therefore, P1A represents a prototype for onco-fetal antigens. To test the potential function of P1A in tumorigenesis, we used a transgenic mouse expressing P1A in lymphoid cells. We observed that immunodeficient host P1A transgenic mice developed thymic tumors after 7 months of age and had shorter survival rates compared to control groups. Most of the 7 examined tumors displayed B cell lineage markers. The P1A transgenic bone marrow cells had higher proliferation ability and more potential progenitors compared to control bone marrow cells. To our knowledge, our data provided the first example that onco-fetal antigen can promote tumorigenesis.

## Introduction

By definition, tumor antigens were identified because cancer-reactive lymphocytes could recognize these proteins [1]. An interesting but still poorly understood phenomenon is that many tumor antigens are also abundantly expressed at early stages of embryogenesis. These antigens are termed onco-fetal antigens. Theoretically, these antigens might be useful for cancer immune therapy, since they are highly expressed on tumor cells but absent in the adult host with the exception of placenta and testis. In addition, the expression of these types of antigens on tumor cells may reflect the fact that cancer may resemble the biological properties of embryonic cells. One key issue is whether the onco-fetal antigens play a significant role in cell differentiation or proliferation, since tumor and embryonic cells share some biological properties.

P1A is a prototype of onco-fetal antigens. Only recently, the P1A gene has been mapped to the X-chromosome (Mouse Genome Informatics, ID 98818). Identification of P1A as a tumor rejection antigen marked an important breakthrough in tumor immunology as it was the first tumor antigen known to be recognized by cytotoxic T cells [2]. P1A was first identified in the mastocytoma P815 cell line which was derived from DBA/2 mice after exposure to methylcholantrene [3]. Later, studies also found that P1A was expressed in several tumors such as Meth A sarcoma and J558 plasmacytoma [4]. P1A is highly expressed in the testis and placenta [5]. The levels of P1A mRNA in the testis and placenta are around 10–20% and 150–200% of mastocytoma P815, respectively [5]. In addition, P1A mRNA was detectable in spleen, lung, liver and thymus by reverse transcription- polymerase chain reaction (RT-PCR), although the relative abundance of P1A mRNA in these tissues was much lower than that found in J558 tumor cells [6]. Here, we show that P1A is also expressed in mouse R1 embryonic stem (ES) cells.

The function of P1A is unknown to date. Since tumor cells, germ cells and stem cells have the capacity to differentiate and proliferate, it is possible that P1A may be involved in a particular state of cell differentiation and growth regulation. Therefore, to seek the function of onco-fetal antigens, we used P1A as a model. In this paper, we found that our transgenic mouse line, which over-expresses tumor antigen P1A in lymphoid cells, developed a variety of tumors after 7 months. The results from this study suggest that P1A is involved in tumorigenesis.

## Results

### P1A express in embryonic stem cells and P1A transgenic (P1A Tg) thymus

**Figure 1:**
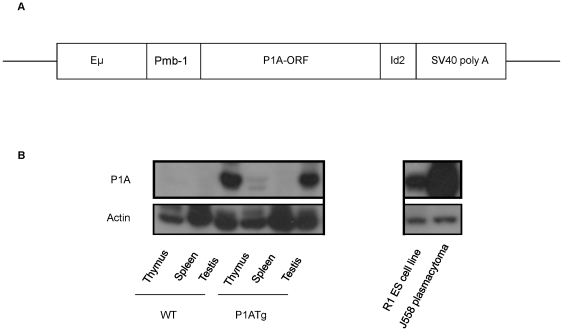
Expression of P1A. A. Diagram of Eμmb-P1A transgene. P1A open reading frame (P1A-ORF) is under controlled by enhancer of immunoglobulin heavy chain (Eμ) and mb-1 promoter (P_mb-1_), followed by ID2 gene and an SV40 polyadenylation signal sequence. B. Western blot analysis of P1A protein expression in J558 plasmacytoma cells, R1 murine ES cells, thymus, spleen, testis from P1A Tg mice and BALB/c WT mice; actin as internal control.

Our P1A transgene contained the enhancer of the immunoglobulin heavy chain gene (Eμ), the mb-1 promoter, followed by P1A open reading frame, an ID2 gene, and an SV40 polyadenylation signal (Figure 1A). P1A was under the control of the mb-1 promoter, which is expressed specifically in B cells from the progenitor to mature stages [7]. In addition, the enhancer of Eμ has been shown to promote transgene expression in B and T lymphoid cells [8], [9], [10], [11], [12]. Thus, our P1A Tg mice can express P1A in lymphoid cells. P1A protein was detectable by Western blot in P1A Tg thymus by the anti-P1A antibody against the C-terminus of the P1A protein, however it was not detected in P1A Tg spleen even with much more protein loaded (Figure 1B). As shown in Figure 1B, P1A protein level was highest in J558 plasmacytoma cells followed by mouse R1 embryonic stem cells and then the testis, according to the actin loading amount.

### P1A Tg mice developed thymic tumors

**Figure 2:**
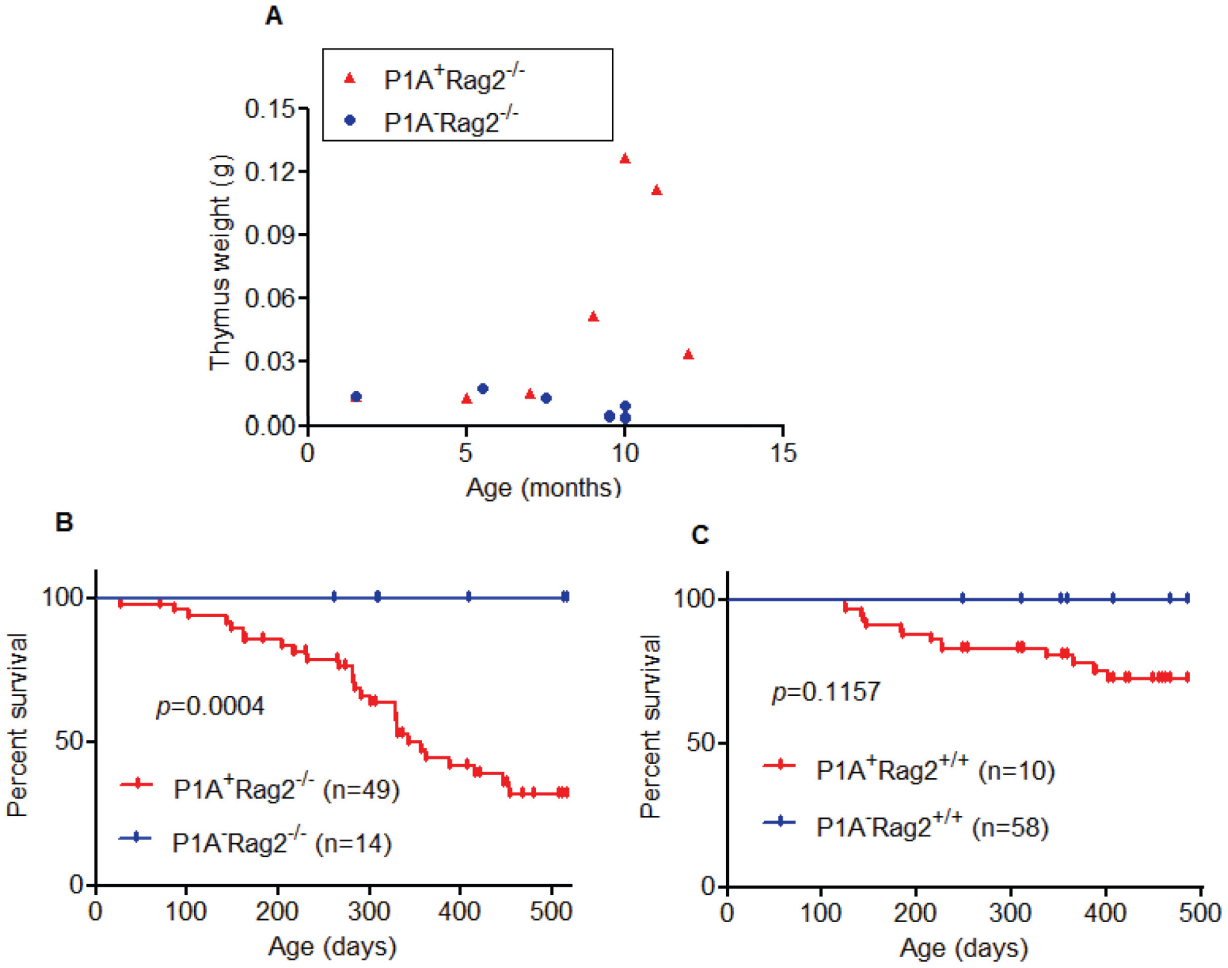
P1A Tg mice developed tumors. A. Thymus weights of *P1A^+^Rag2^−/−^* mice and Rag2^−/−^ mice. Enlarged thymus in P1A^+^Rag2^−^ mice after 7 months of age compared to P1A^+^Rag2^−^ mice. B. P1A^+^Rag2^−^ mice had shorter survival rates compared to P1A^−^Rag2^−^ groups. Only mice that died due to tumor formation were recorded. *p* = 0.0004 (Kaplan-Meier analysis). C. Although several P1A^+^Rag2^+^ mice died due to tumor formation, differences in the tumor incidences were not statistical significant. *p* = 0.1157 (Kaplan-Meier analysis).

To increase tumor susceptibility, we crossed the P1A transgene to *Rag2^−/−^* background which is immune-deficient due to lack of T and B lymphocytes. We observed that 19 out of 49 P1A Tg mice in Rag2-deficient background developed thymic lymphoma after 7 months of age. The Rag2^−/−^P1ATg mice had enlarged thymuses compared to control mice (Figure 2A). In addition to the thymus, the spleen was also enlarged (data not shown). Moreover, tumor infiltration into target organs, including liver and lung, were also widespread (data not shown). In addition, when using tumor incidence as a survival end point, P1A Tg mice in immunodeficient background (Rag2^−/−^) had significant shorter survival rates due to the tumor formation compared to control groups (*p* = 0.0004, Figure 2B). A trend of increased tumor was also noted in WT background, although the increase was not statistically significant (*p* = 0.1157, Figure 2C).

**Figure 3:**
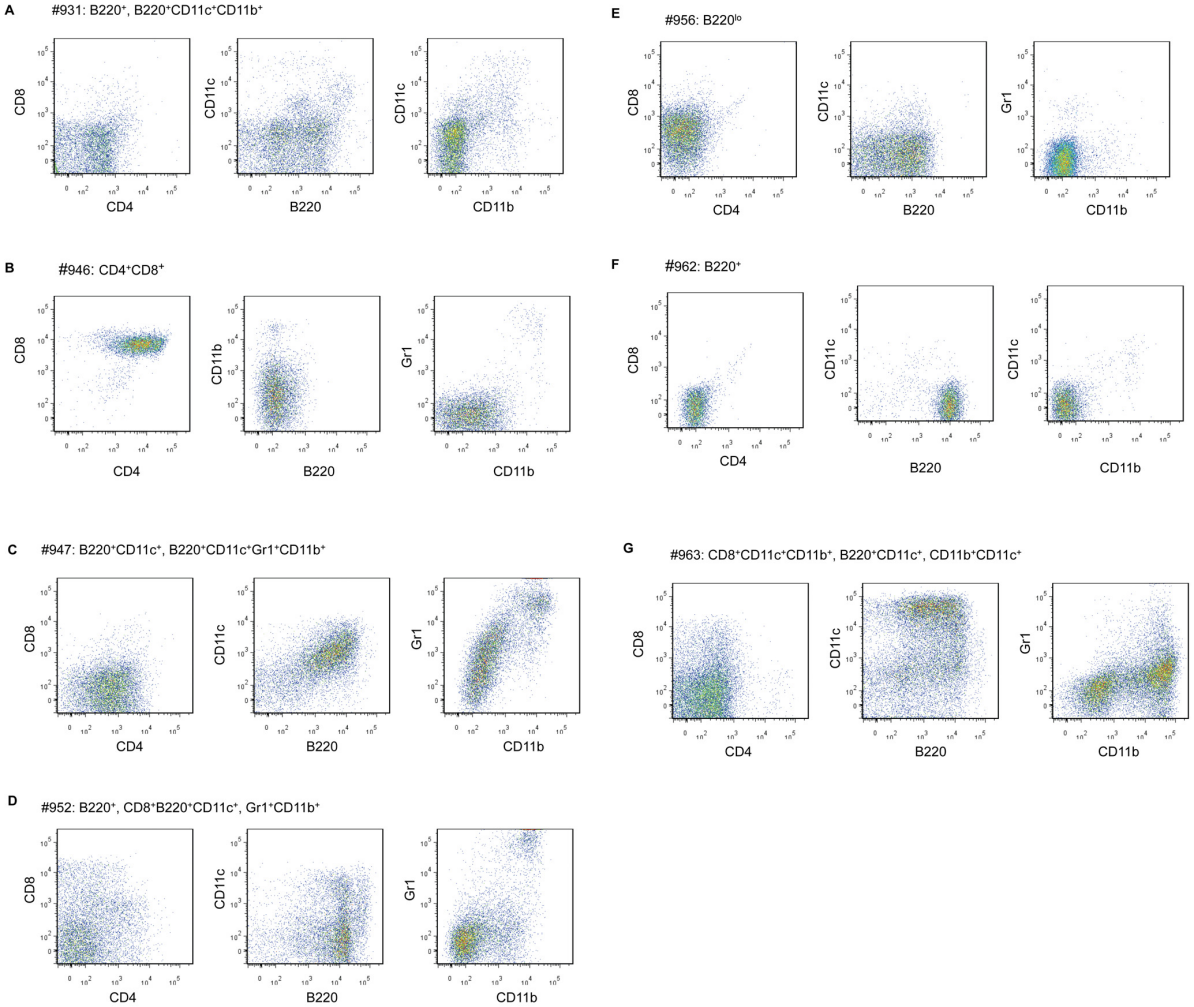
Heterogeneity of tumors in individual mouse. The thymic tumor from different mice was stained by the following monoclonal antibodies, CD4, CD8, B220, CD11c, Gr1, and CD11b. After antibody staining, thymic tumor cells were subsequently analyzed by flow cytometry. A. Mouse #931 had thymic tumors with B220^+^ and B220^+^CD11c^+^CD11b^+^ cell populations of tumor cells. B. Mouse #946 had thymic tumor of CD4^+^CD8^+^ cell population of tumor cells. C. Mouse #947 had B220^+^CD11c^+^ and B220^+^CD11c^+^Gr1^+^CD11b^+^ cell populations of tumor cells. D. Mouse #954 had B220^+^, CD8^+^B220^+^CD11c^+^, Gr1^+^CD11b^+^ cell populations of tumor cells. E. Mouse #956 thymic tumor had B220^lo^ cell population of tumor cells. F. Mouse #962 had tumor with B220^+^ cell population of tumor cells. G. Mouse #963 showed CD8^+^CD11c^+^CD11b^+^, B220^+^CD11c^+^ and CD11b^+^CD11c^+^ cell populations of tumor cells.

In order to determine the lineages of the tumors, we analyzed the cell surface markers of the thymic tumors. As shown in Figure 3, only 1 of the 7 tumors analyzed expressed significant levels of CD4 and CD8. In contrast the majority of tumors expression markers of non-T lineages, including that of B cells (B220^+^), plasmacytoid dendritic cells (B220^+^CD11c^+^) , myeloid cells (B220^+^Gr1^+^CD11b^+^). The result is consistent with the fact that Rag2 deficiency results in the thymocyte differentiation being blocked before they can reach the CD4^+^CD8^+^ stage [13]. It is noteworthy that all but one of tumors expresses B220, a marker for the B cell lineage.

### P1A Tg bone marrow (BM) cells exhibit higher proliferation and more hematopoietic colony forming units

**Figure 4:**
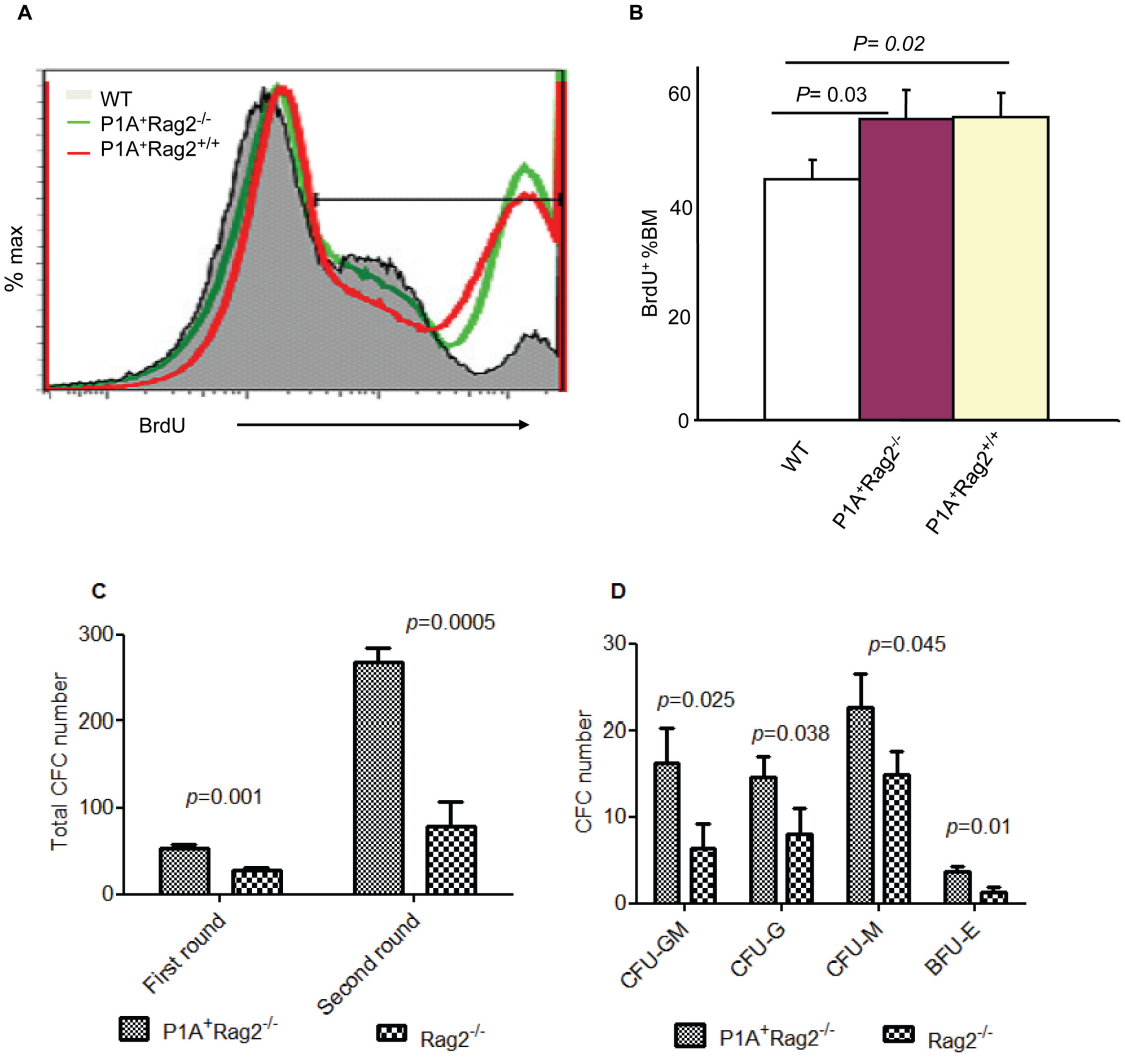
Enhanced proliferation and progenitor activity of P1A Tg bone marrow. A. BrdU incorporation by flow cytometry; representative profiles of BrdU incorporation in bone marrow cells. B. The proportion of BrdU positive cells among bone marrow cells, showing mean ± SD of BrdU positive cells (n = 3, two-tailed unpaired Student's t-test). C. Colony-forming cell (CFC) assay. There was a higher colony number in P1A^+^Rag2^−^ compared to Rag2^−^ bone marrow cells. The cells cultured in second round were taken from the first round culture (n = 3, two-tailed unpaired Student's t-test). D. Higher colony number in all colony types, including burst-forming unit-erythroid (BFU-E), colony-forming unit granulocyte (CFU-G), colony-forming unit macrophage (CFU-M) and colony-forming unit granulocyte macrophage (CFU-GM), were seen in P1A^+^Rag2^−^ than in Rag2^−^ by inverted microscopy. (n = 3, two-tailed unpaired Student's t-test)

We determined P1A Tg BM cell proliferation ability by BrdU incorporation assay. BrdU is an analog of the DNA precursor thymidine. The proliferating cells will incorporate BrdU into their DNA. The amount of BrdU in the DNA of cells can be detected with specific anti-BrdU fluorescent antibodies followed by flow cytometry. After 24 hours of labeling BrdU in vivo, we analyzed the BrdU incorporation rate by flow cytometry. Forty-six percent of BM cells from wild-type (WT) control mice were BrdU positive. However, the P1A Tg mice had a significant increase in the proportion of BrdU-positive BM cells both in immunocompromised (P1A^+^Rag2^+/+^) and immunodeficient (P1A^+^Rag2^−/−^) backgrounds compared to WT mice (*p* = 0.02 and 0.03, respectively, Figure 4 AB).

We also cultured P1A Tg BM cells and control cells in methylcellulose based medium to quantify potential progenitors in vitro. The hematopoietic progenitors were able to proliferate and differentiate, resulting in the generation of mature cells. Colony-forming units (CFUs) were measured in semi-solid media with appropriate cytokines. We found P1A Tg BM had much higher CFU compared to control BM. The increase was more obvious in the second round culture (Figure 4C). According to their morphology, the increases of colony number in all colony types, including BFU-E (burst-forming unit-erythroid), CFU-G (colony-forming unit granulocyte), CFU-M (colony-forming unit macrophage) and CFU-GM (colony-forming unit granulocyte macrophage), were seen in P1A Tg BM by inverted microscopy (Figure 4D).

## Discussion

P1A, the first identified tumor rejection antigen, is presented to cytotoxic T lymphocytes by bound to major histocompatibility complex (MHC) class I molecules on the tumor cell surface [14]. Our data on the expression of P1A in tumor cells and its presence in embryonic stem cells and testis indicated that P1A is an onco-fetal antigen. In testis, it has been shown that P1A expression is restricted to spermatogonia cells which are MHC class I negative cells [5]. In addition, MHC class I molecules are not detected in the undifferentiated ES cells by FACS or immunofluorescence staining in ES cells [15]. Hence, the low to no expression of cell-surface MHC class I on spermatogonia and ES cells suggests that they are immune privilege. Therefore, these normal cells expressing P1A antigen are compromised in the immune system due to low MHC class I expression, but not tumor cells.

The phenotypes of P1A Tg mice suggested that onco-fetal antigen P1A was indeed involved in tumorigenesis. First, we observed that P1A Tg mice developed thymic leukemia after 7 months of age, particularly in immunocompromised mice. Second, we demonstrated higher BrdU incorporation in P1A Tg BM cells compared to WT cells in vivo. Third, there was more CFU from P1A Tg BM compared to control in vitro. To our knowledge, this is the first evidence that P1A may promote tumorigenesis, and the first example that onco-fetal antigen can be involved in tumorigenesis.

Mice with inactivated *Rag2* cannot rearrange lymphocyte antigen receptors and therefore lack peripheral T cells and B cells [13], [16]. In thymus, after TCR-β gene rearrangement, the CD4^−^CD8^−^ double negative cells differentiate into CD4^+^CD8^+^ double positive thymocytes which are selected to single-positive cells (CD4 or CD8) following TCR-α rearrangement. As a result, T cell development in Rag2-deficient thymus is arrested prior to the CD4^+^CD8^+^ stage. The appearance of a thymic tumor at CD4^+^CD8^+^ stage suggest that transformation of T cells may have resulted in expression of the CD4 and CD8 molecules. The role for P1A in the process is unknown. Likewise, B cell development in the Rag2-deficient mice is blocked at the pro-B cell stage which could express cell surface marker, B220 [13], [16]. The widespread expression of B220 among the tumors is therefore anticipated. However, the occurrence of thymic tumor in all tumor-bearing mice deserves consideration. CD4^−^CD8^−^ double negative thymocytes contain a mixture of lineage-committed progenitors which can reconstitute T, B, NK and dendritic cell lineages [17], [18], [19]. Our transgene P1A was expressed during hematopoietic progenitor development; therefore, any stage of cells may be the targets of the transformation or immortalization process by P1A activation. The heterogeneity was probably the result of tumor initiation at different stage of progenitor cell development. Hence, P1A induced tumorigenesis is not stage-specific and resulted in various thymic tumor types in mice, such as B cells (B220^+^), plasmacytoid dendritic cells (B220^+^CD11c^+^) and myeloid cells (Gr1^+^CD11b^+^).

Several clinical studies have indicated that the frequency of cancer in immunodeficient background is more frequent [20], [21], [22]. For example, children with immunodeficiency are at a higher risk of malignancy, such as lymphomas and leukemias [23]. Therefore, the immune system, including innate and adaptive immunity, is able to inhibit the tumor development and eradicate the tumors already established [21]. A similar phenomenon was also shown in animal models. For example, after subcutaneous injection of the chemical carcinogen methylcholanthrene, *Rag2^−/−^* background mice developed higher frequency of sarcomas compared to the strain-matched controls [24]. Our data indicated that thymic tumors occurred more frequently in the Rag2-deficient background and the immune system of a normal immunocompetent mouse may be able to eliminate the P1A-induced tumor.

Taken together, P1A induced tumorigenesis and resulted in various thymic tumor types in mice. Our present work demonstrated that onco-fetal antigen P1A was associated with the transformed phenotype in P1A transgenic mice. Therefore, expression of onco-fetal antigens may be biologically relevant to tumorigenesis. Further studies are required to dissect the mechanisms of the process of oncogenesis by P1A.

## Materials and Methods

### Cell lines and experimental animals

Mouse plasmacytoma J558 from ATCC were cultured in RPMI 1640 medium containing 5% fetal bovine serum (FBS) and 100 µg/ml penicillin and streptomycin. Murine R1 ES cells were co-cultured with mouse embryonic fibroblast (MEF) cells in DMEM medium containing 15% FBS, 0.1 mM β-mercaptoethanol, 10^3^ u/ml leukemia inhibitory factor, and 4 mM glutamine. Rag2^−/−^ mice were obtained from the Taconic Laboratories (Terrytown, NY). P1A transgenic (P1A Tg) mice were as described previously [6], [9]. The transgene has been crossed to BALB/c for more than 10 generations, and was crossed to BALB/c *Rag2^−/−^* background for the current studies. P1A^+^Rag2^−/−^ mice in this study came from 7 breeding pairs. All animal experiments were conducted in accordance with accepted standards of animal care and approved by the Institutional Animal Care and Use Committee of University of Michigan.

### Western blot analysis

Samples of mouse tissues or cells were lysed in protein lysis buffer (50 mM Tris-HCl, pH 7.4, 150 mM NaCl, 0.5% NP-40) and protease inhibitor cocktails (Sigma) including 4-(2-aminoethyl) benzenesulfonyl fluoride hydrochloride, aprotinin, bestatin, E-64, leupeptin and pepstain A were added. Cell lysates were separated by 10% sodium dodecyl sulphate-polyacrylamide gel electrophoresis, transferred to polyvinylidene fluoride (PVDF) membranes, and incubated with anti-P1A rabbit antibody specific for the peptide sequence, YEMGNPDGFSP (Genemed Synthesis, 1∶500 dilution) or anti-actin mouse antibody (Sigma, 1∶5000 dilution). Anti-rabbit or anti-mouse IgG horseradish peroxidase–linked antibody at 1∶3500 dilutions (GE Healthcare) was used as secondary antibodies. Antibodies were detected with chemiluminescence reaction using the enhanced chemiluminescence kit (Amersham Biosciences) and visualization with exposure to film.

### Flow cytometric analysis

P1A transgenic mice were sacrificed when moribund. Thymus and spleen tissue were homogenized and passed through a cell strainer (BD Biosciences) to generate a single-cell suspension. Cells were stained in phosphate-buffered saline plus 2% FBS for 20 minutes on ice with the following monoclonal antibodies at a 1∶200 dilution, CD4, CD8, B220, CD11c, Gr1, and CD11b (BD Biosciences). Cells were subsequently analyzed by BD LSR II Flow Cytometer.

### Bromodeoxyuridine (BrdU) incorporation assay

Mice were injected intraperitoneally with BrdU (100 mg/kg, Sigma-Aldrich). Then, mice were given BrdU water (1 mg/ml) for 24 hours before sacrifice. BrdU staining kit (BD Biosciences) was used according to the manufacturer's instructions. Each group had three mice from two independent experiments.

### Colony-forming cell assay

Bone marrow cells were isolated from the femur and tibia of mice. Bone marrow cells (1×10^4^) were plated in MethoCult^®^ methylcellulose-based media (StemCell Technologies) per 35mm dish and incubated at 37°C, 5% CO_2_. After 12 days culture, colony number and morphology was counted using inverted microscope. Bone marrow cells were from one Rag2^−/−^ and one P1A^+^Rag2^−/−^ mouse and each group was tested in triplicates from a single experiment.

### Statistics

The differences in mouse survival rates were calculated by Kaplan-Meier analysis. Other data were analyzed using 2-tailed unpaired Student's t-test. Differences of *p*<0.05 were considered statistically significant. All statistics were performed using GraphPad Prizm, version 5 (GraphPad Software, San Diego, CA).
